# Attention-based multi-scale convolution and conformer for EEG-based depression detection

**DOI:** 10.3389/fpsyt.2025.1584474

**Published:** 2025-07-01

**Authors:** Ze Yan, Yumei Wan, Xin Pu, Xiaolin Han, Mingming Zhao, Haiyan Wu, Wentao Li, Xueying He, Yunshao Zheng

**Affiliations:** ^1^ Key Laboratory of Computing Power Network and Information Security, Ministry of Education, Shandong Computer Science Center (National Supercomputer Center in Jinan), Qilu University of Technology (Shandong Academy of Sciences), Jinan, China; ^2^ Shandong Engineering Research Center of Big Data Applied Technology, Faculty of Computer Science and Technology, Qilu University of Technology (Shandong Academy of Sciences), Jinan, China; ^3^ Shandong Provincial Key Laboratory of Industrial Network and Information System Security, Shandong Fundamental Research Center for Computer Science, Jinan, China; ^4^ Department of Psychiatry, Shandong Mental Health Center, Shandong University, Jinan, China; ^5^ School of Medical Information Engineering, Shandong University of Traditional Chinese Medicine, Jinan, China

**Keywords:** depression detection, electroencephalography (EEG), attention, deep learning (DL), AMCCBDep

## Abstract

Depression is a common mental health issue, and early detection is crucial for timely intervention. This study proposes an end-to-end EEG-based depression recognition model, AMCCBDep, which combines Attention-based Multi-scale Parallel Convolution (AMPC), Conformer, and Bidirectional Gated Recurrent Unit (BiGRU). The AMPC module captures temporal features through multiscale convolutions and extracts spatial features using depthwise separable convolutions, while applying the ECA attention mechanism to weigh key channels, enhancing the model’s focus on crucial electrode channels. The Conformer module further captures both global and local temporal dependencies in EEG signals to ensure the capture of long-range dependencies and local patterns. The BiGRU module improves the model’s ability to recognize depressive states by utilizing bidirectional modeling. We used the 128-channel resting-state EEG signals from the MODMA dataset, which includes data from 24 depression patients (13 males, 11 females, aged 16 to 56) and 29 healthy individuals (20 males, 9 females, aged 18 to 55). Experimental results show that the AMCCBDep model achieved an accuracy of 98.68% ± 0.45% on the MODMA dataset. The model evaluation results for both 128-channel and 16-channel configurations demonstrate that reducing the number of electrodes has a minimal impact on performance, suggesting that electrode reduction could be considered in practical applications. This model showcases strong potential in advancing depression detection in neuroscience, providing an efficient and scalable solution for clinical and practical applications. Future research will further optimize model performance and explore the impact of reducing the number of electrodes on clinical practice.

## 1 Introduction

Depression is a severe mental disorder characterized by persistent sadness, hopelessness, and a lack of interest in most activities, along with changes in sleep, appetite, and difficulty concentrating, as defined by the Diagnostic and Statistical Manual of Mental Disorders, Fourth Edition (DSM-V). It often coexists with anxiety disorders, substance use disorders, and physical health issues like cardiovascular diseases (1). The World Health Organization (WHO) predicts that by 2030, depression could become the leading cause of non-natural deaths globally (2). Therefore, early detection and diagnosis of depression are crucial. However, existing clinical diagnostic methods encounter issues such as susceptibility to misdiagnosis, subjective denial, and low sensitivity (3). Therefore, there is a critical need for a simple, precise, and dependable approach to aid clinicians in diagnosing depression.

The identification of depression primarily relies on scales and interviews, with the final diagnosis typically based on the subjective judgment of experienced psychiatrists. However, there is currently a lack of effective and objective assessment criteria for depression in clinical practice (4). In recent years, with the advancement of science, technology, and healthcare in China, neuroimaging technologies such as electroencephalography (EEG) have seen increasing application in depression research. Compared to other technologies, EEG can capture neural electrical activity in the brain with millisecond temporal resolution, offering high reliability and low cost (5). However, EEG signals have relatively low spatial resolution, which limits their application in capturing detailed brain activity. Additionally, other imaging technologies such as computed tomography (CT) (6) and magnetic resonance imaging (MRI) are also widely used in the diagnosis of acute neurological disorders. CT provides higher spatial resolution, while methods like functional MRI (fMRI) can improve diagnostic accuracy by extracting nonlinear factor matrices from data using deep learning techniques such as Deep CSAF (7) and HB-DFL (8), without relying on prior knowledge (9). The Deep WTFAF model has also been proposed, which enhances the key features of fMRI data through a time-frequency attention module, offering new perspectives for neuroimaging analysis.

Additionally, EEG technology has been widely applied in the diagnosis of other psychiatric disorders, particularly in depression research. Alaei et al. (10) employed EEG source reconstruction and graph theory-based directed brain network analysis to investigate the differences between anxious depression and non-anxious depression patients. They found significant differences in brain network connectivity strength and betweenness centrality in anxious depression patients. These findings provide new directions for the subtyping of depression. Aydın et al. (11) studied the brain network indices in boys with ADHD-C and found that long-term medication treatment significantly improved brain functional connectivity, particularly in increasing the brain’s segregation and resilience. This study offers new insights into the application of EEG in childhood psychiatric disorders. Knott et al. (12) revealed abnormalities in interhemispheric synchrony and asymmetry in the brains of depression patients. These findings provide important biological markers for understanding the EEG characteristics of male depression.

The progress of artificial intelligence has enabled automated solutions for diagnosing mental disorders (13). In the area of brain-computer interfaces, brainwave signal feature extraction networks are progressively evolving to deeper levels and are applied to tasks such as motor imagery, sleep staging, and emotion recognition, with depression recognition being no exception. In recent years, with advancements in deep learning for computation, this technology has become increasingly popular in the field of depression recognition and has been widely applied. For example, Su et al. (14) introduced the 3DMKDR model, which enhances depression recognition accuracy by transforming EEG signals into three-dimensional structures and utilizing multi-scale convolution kernels. Chen et al. (15) introduced LG-GCN, a graph convolutional network that combines local and global graph representations. Through multi-graph fusion and information enhancement modules, it improves the spatial feature extraction capabilities of EEG data. Zhang et al. (16) proposed a spatial-temporal EEG fusion method based on neural networks for depression detection, using LSTM to extract time-domain features, constructing brain functional networks with PLI, and extracting spatial-domain features via 2D CNN, followed by feature fusion. Since EEG signals are one-dimensional, extending them into three-dimensional or four-dimensional space for feature extraction in the aforementioned methods may lead to overfitting and excessively long training times. Additionally, multi-scale information is crucial for EEG signals, as many physiological components operate on different time scales (17, 18), yet most existing depression recognition methods do not focus on this aspect. The concept of Transformer is considered one of the most powerful concepts in deep learning today, and it is also widely applied in EEG classification. Wang et al. (19) proposed a Transformer-based model, which enhances the ability to capture global dependencies in EEG by integrating information at the electrode and brain region levels while emphasizing key brain regions, although it overlooks the importance of local features. Furthermore, RNNs have been widely used in EEG analysis due to their excellent time-series data processing capabilities. For example, in recent research, Luo et al. (20) combined graph neural networks (GNN) and gated recurrent units (GRU) to capture the spatiotemporal dependencies of brain networks and introduced a distance-based universal graph adjacency matrix and a learnable correlation matrix to model individual brain network differences.

To tackle the previously mentioned challenges and inspirations, this paper introduces a hybrid model for depression recognition, which combines Attention-based Multi-Scale Parallel Convolution (AMPC), Conformer, and Bidirectional Gated Recurrent Units (BiGRU). The AMPC module extracts rich time-frequency features from different dimensions through multi-scale convolution and the ECA mechanism. The Conformer module combines self-attention and convolution, enabling the model to capture long-range global dependencies while also processing local time-series features. The BiGRU module uses a bidirectional recurrent neural network to capture bidirectional dependencies along the time dimension, enhancing the model’s capacity to represent time-series data. The main contributions of this paper are summarized as follows:

- Firstly, a hybrid model based on attention-based multi-scale Parallel convolution is proposed, which also incorporates the Conformer encoder and Bidirectional Gated Recurrent Units (BiGRU) to handle the feature extraction and classification of EEGsignals in a seamless, end-to-end process.
- Secondly, by extracting features from multiple scales and utilizing attention to adaptively focus on key channels, the model’s feature representation ability and computational efficiency are enhanced, effectively improving the accuracy and robustness in processing complex data.
- Finally, results from experiments on the MODMA public dataset show that the proposed model excels in classification accuracy and F1 score. Additionally, ablation studies confirm the model’s effectiveness.

The structure of the rest of the paper is as follows. Section 2 describes the materials and methods. The experimental results are presented in Section 3. Section 4 provides a discussion of the proposed method. Finally, Section 5 concludes the paper.

## 2 Materials and methods

### 2.1 Dataset

The EEG data used in this study includes 128-channel resting-state signals from the MODMA dataset, a multimodal open resource designed for mental disorder analysis (21, 22). This dataset includes 24 patients with depression (13 males and 11 females, aged 16 to 56) and 29 healthy individuals (20 males and 9 females, aged 18 to 55). EEG data were recorded while participants were awake with their eyes closed, seated in a quiet, soundproof, and well-ventilated room, maintaining a stationary position without any head or limb movement and without unnecessary eye movements (e.g., scanning, blinking, etc.). Data collection was carried out using the Net Station software and the HydroCel Geodesic Sensor Net (HCGSN) (Electrical Geodesics Inc., Oregon Eugene, USA), version 4.5.4. The sampling frequency was set at 250 Hz, with the Cz electrode as the reference point, and approximately 5 minutes of EEG signal data was recorded for each participant. All participating patients with depression met the diagnostic standards for depression as defined by the Diagnostic and Statistical Manual of Mental Disorders, Fourth Edition (DSM-IV) (23).

### 2.2 Preprocessing

Due to the high sensitivity of EEG signals to external environments and other physiological signals within the body, their raw data is often subjected to various types of interference before processing. To resolve this problem, we applied a set of preprocessing steps to the EEG data. Inspired by previous work (24), Firstly, considering both time efficiency and computational cost, this study selected 16 electrodes (Fp1/2, F3/4, C3/4, P3/4, O1/2, F7/8, T3/4, T5/6) from the original 128 electrodes. Previous studies have demonstrated the feasibility of using these electrodes to identify depressive states. Akar et al. (25) emphasized the significance of the frontal and parietal regions in emotion regulation and cognitive control. Abnormal activity in these regions is frequently observed in depression, making them highly suitable for detecting depression-related EEG signals. The 16 selected electrodes cover key brain areas involved in emotional processing, cognitive control, and self-regulation. Li et al. (26) found that EEG signals from the parietal and frontal regions are crucial for detecting mild depression, and that these regions enhance classification accuracy. These areas are essential for cognition and emotional regulation, and selecting electrodes from these regions is supported by solid evidence. Sun et al. (27) highlighted the importance of functional connectivity features, such as the Phase Lag Index (PLI), in depression recognition, particularly intrahemispheric connections. The selected electrodes (e.g., F3, F7, T3) are located in these critical brain regions and capture functional connectivity data that may reveal significant differences in brain activity associated with depression. According to Aydın et al. (28), emotion regulation is linked to spectral coherence in EEG signals, with the frontal and parietal regions playing a central role. Thus, we selected electrodes from these regions, including the frontal (F) and parietal (P) regions, as well as the occipital (O) region. These brain areas are integral to emotional processing and are often dysregulated in individuals with depression, making them highly relevant for our analysis. Next, a notch filter was used to remove 50Hz power line interference, and a band-pass FIR filter was applied to preserve the frequency range between 1Hz and 40Hz. Following that, the EEG data was divided into 4-second windows, with a 75 % overlap between adjacent windows (29). Fourth, the automatic artifact rejection algorithm Autoreject (30) was used to generate cleaner EEG data. Fifth, normalization was performed on the data, which was processed using a functional normalization method before entering the network structure, as shown in Equation 1:


(1) $$z=\frac{{\text{X}}_{i,j}-\mu }{\sigma },$$

Here, $X_{i,j}$ represents the feature vector, μ denotes the mean of the feature vector, and σ represents the standard deviation of the feature vector.

### 2.3 Proposed methodology

**Figure 1:**
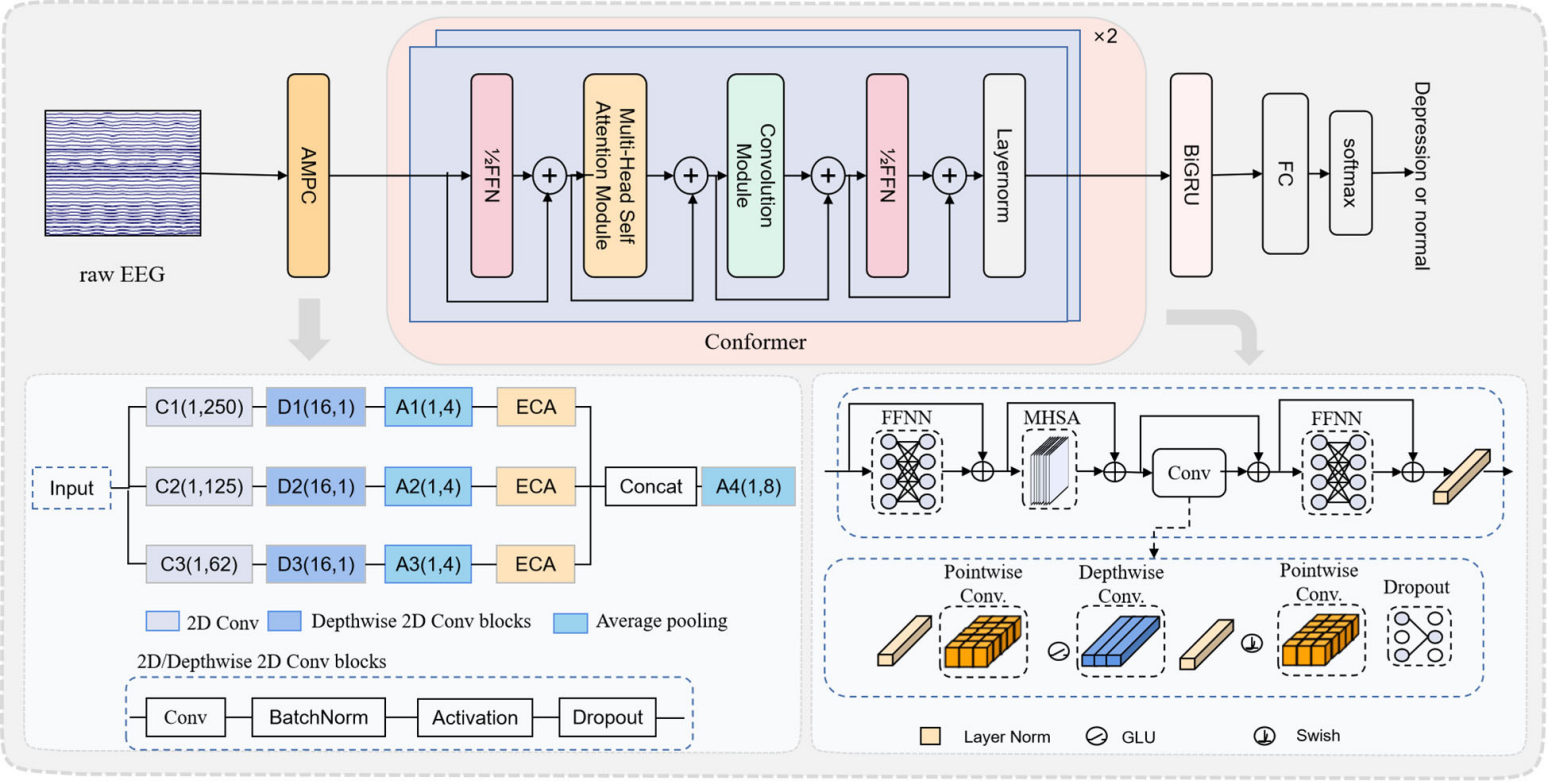
The deep learning framework diagram of the AMCCBDep model for depression recognition based on EEG signals. The framework consists of three main components: the AMPC module, the Conformer module, and the BiGRU and Classification.


The research framework we proposed is depicted in **Figure 1**. Following data preprocessing, we developed the AMCCBDep model to tackle the depression classification task. This section outlines the architectural design of AMCCBDep, which comprises three key modules: the AMPC module, the Conformer module, and the BiGRU module.

#### 2.3.1 AMPC module

As shown in **Figure 1**, inspired by the neurophysiological characteristics of EEG signals and the work in (24), this paper designs an attention-based multi-scale parallel convolution (AMPC) module to extract network modules for multi-level frequency features. The module sets the size of the temporal convolution kernel *H* to a specific ratio of the EEG sampling frequency *f_s_*. These ratios are defined as *α*. Therefore, *S_T_* represents the size of *H*, as shown in Equation 2:

(2) $$S_{T}=\left(\begin{array}{cc} 1, & \alpha \cdot \text{fs} \end{array}\right)$$

At the same time, excessive convolution operations increase the number of parameters, which can affect computational efficiency. Therefore, without affecting classification performance, the time convolution layer is designed with three branches. The ratio coefficients for the parallel convolution layers range from 1 to 3, and are set to [1,0.5, 0.248], corresponding to the sizes of *H* as [(1,250),(1,125),(1,62)]. From a frequency perspective, when the length of *H* is set to different ratios of the sampling frequency *f_s_*, the time convolution layer can capture frequencies of 1 Hz, 4 Hz, and 8 Hz and above. From a temporal perspective, these kernel sizes correspond to time steps of 1000 ms, 248 ms, and 124 ms, respectively.

**Figure 2:**
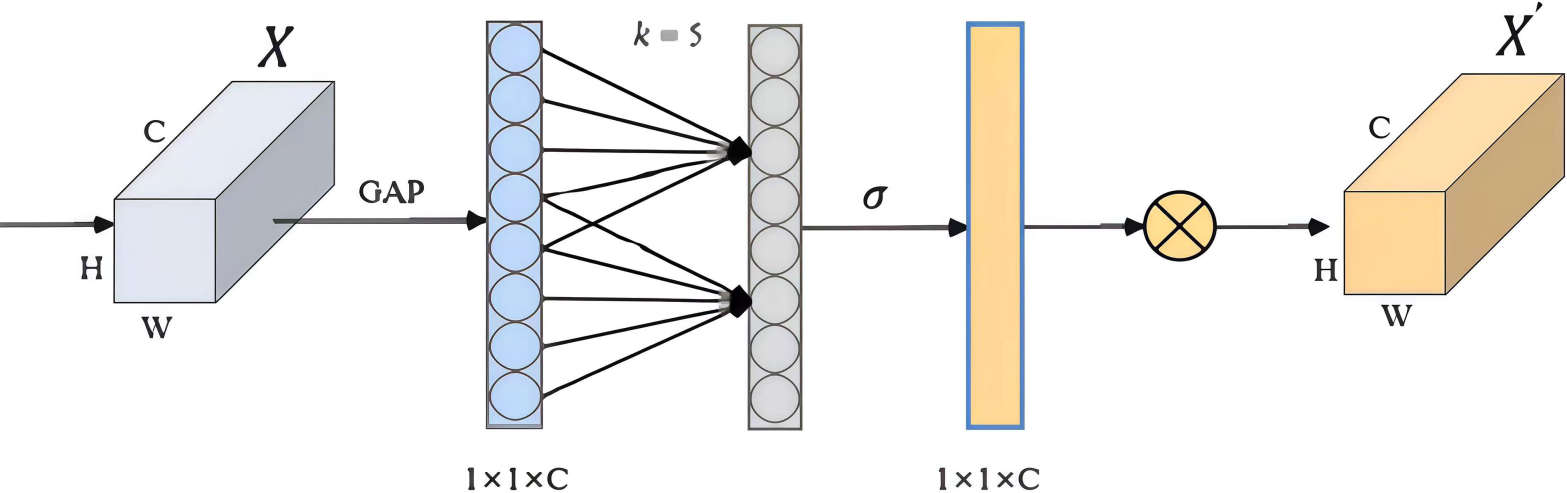
ECA module configuration.

The AMPC module extracts EEG features by applying three types of convolutions in parallel. First, 2D convolution performs frequency filtering along the time axis. Second, deep convolution processes spatial features, followed by an average pooling layer to reduce the time dimension, with dropout applied to prevent overfitting. Next, the ECA attention mechanism (31) is introduced in each branch. The ECA attention mechanism adaptively assigns weights to different EEG channels, enhancing the model’s focus on key channels, thus better capturing depression-related features. In time-series data, higher weights are typically assigned to important features, while less relevant features are given lower weights. Therefore, the ECA attention mechanism prioritizes processing relevant information, enhancing the network’s ability to identify and respond to critical features. The configuration of the ECA module is shown in **Figure 2**.

In the ECA module, channel weights are generated by applying a 1D convolution with creatively selected kernel sizes (*k*) on the data aggregated through Global Average Pooling (GAP). The value of *k* is determined by mapping *C*.

Finally, the outputs from each branch are fused in the concatenation layer, followed by downsampling through an average pooling layer with a kernel size of (1, 8) to improve computational efficiency.

#### 2.3.2 Conformer module

This paper introduces the Conformer encoder (32) because Transformer models are effective at capturing global, content-based interactions, while CNNs excel at leveraging local features. To combine the strengths of both, a parameter-efficient approach is used to model both local and global dependencies within sequences. As illustrated in **Figure 1**, the Conformer module incorporates two half-step feedforward neural networks (FFNNs). Between these FFNNs, multi-head self-attention (MHSA) with four heads is applied, followed by a convolutional module. The convolutional module in the figure begins with layer normalization, a pointwise convolution layer, and a gated linear unit (GLU) activation to address the vanishing gradient problem. The GLU output is then processed through a 1D depthwise convolution layer with a Swish activation function, followed by another pointwise convolution layer. A dropout layer is applied at the end to regularize the network. The computation for the Conformer is as shown in Equation 3:


(3) $$\begin{array}{l} {\tilde{x}}_{i}=x_{i}+\frac{1}{2}\text{FFN}\left(x_{i}\right)\\ x_{i}^{'}=\tilde{x_{i}}+\text{MHSA}\left(\tilde{x_{i}}\right)\\ x_{i}^{''}=x_{i}^{'}+\text{Conv}\left(x_{i}^{'}\right)\\ y_{i}=\text{Layernorm}\left(x_{i}^{''}+\frac{1}{2}\text{FFN}\left(x_{i}^{''}\right)\right) \end{array}$$

FFN refers to the Feed-Forward Network module, MHSA denotes the Multi-Head Self-Attention module, and Conv represents the Convolution module.

#### 2.3.3 BiGRU module

**Figure 3:**
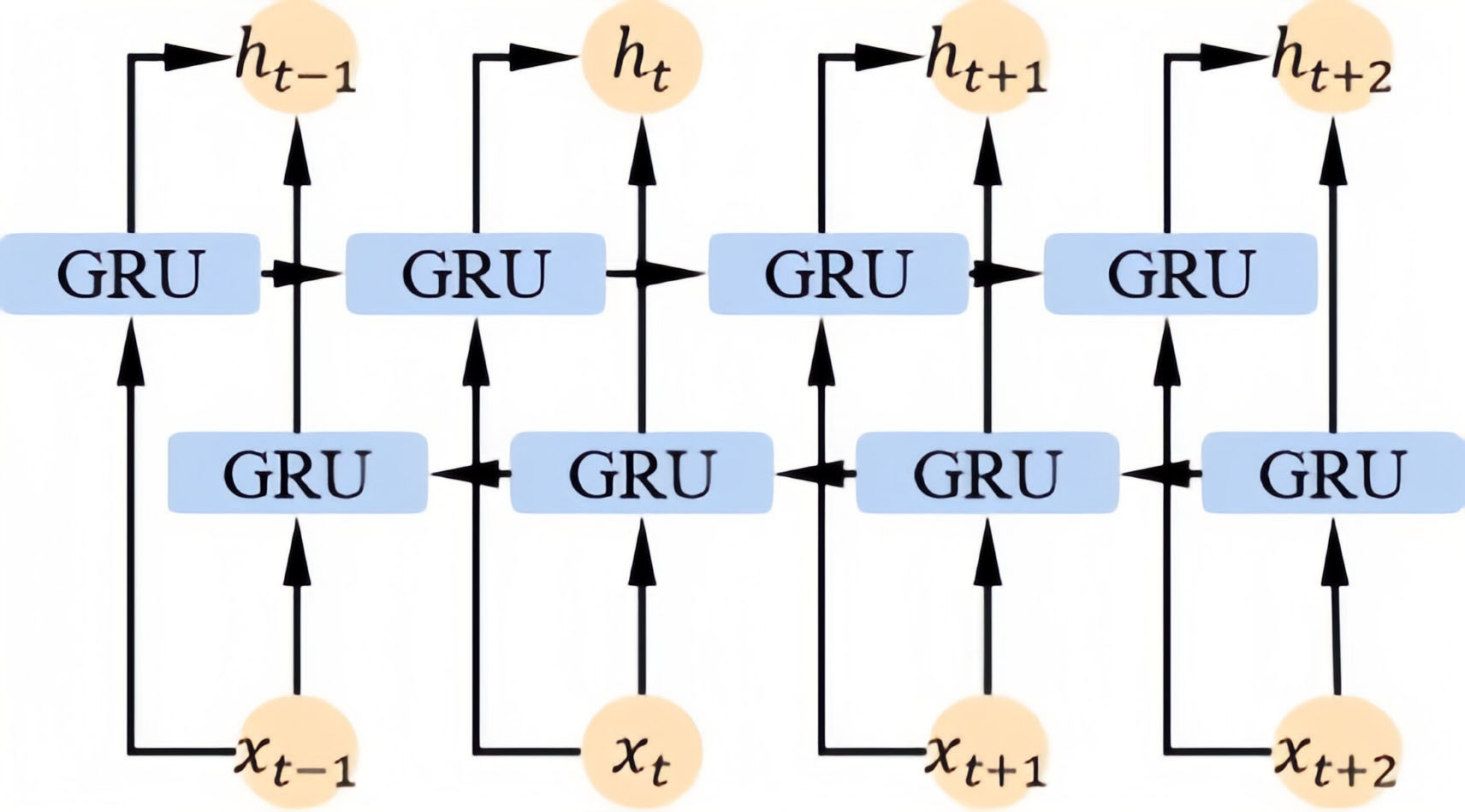
BiGRU module configuration.

Since EEG signals contain dynamic content, variations between time slices may conceal additional information that could contribute to more accurate depression classification. Therefore, we utilize a BiGRU to extract contextual information from the output of the conformer. As illustrated, the basic unit of the BiGRU consists of two GRU cells: one for forward propagation and the other for backward propagation. The configuration of the BiGRU module is shown in **Figure 3**.

The BiGRU module combines forward and backward GRUs to capture contextual information from both past and future time steps. Its operations primarily include forward propagation, backward propagation, and hidden state updates. The corresponding equations are as shown in Equation 4:


(4) $$\begin{array}{l} \vec{h_{t}}=GRU\left(x_{t},\vec{h_{t-1}}\right)\\ \overset{\leftarrow }{h_{t}}=GRU\left(x_{t},\overset{\leftarrow }{h_{t+1}}\right)\\ h_{t}=\left[\vec{h_{t}},\overset{\leftarrow }{h_{t}}\right] \end{array}$$

#### 2.3.4 Classification

The output from the BiGRU is fed into the fully connected layer, and the final classification result is produced using the softmax function. In deep learning models, the main purpose of the softmax function is to transform the network’s output into a probability distribution, ensuring each output value lies between 0 and 1, with the sum of all probabilities equaling 1. The softmax formula is as shown in Equation 5:


(5) $$y=\text{softmax}\left(W_{\circ }h_{\text{f}}+b_{\circ }\right)$$

Here, *h_f_* denotes the output of the BiGRU module, *W*_°_ represents the weight matrix of the fully connected layer, and *b*_°_ is the bias term of the fully connected layer.

### 2.4 Evaluation

The evaluation metrics used in this study include classification accuracy, sensitivity, specificity and F1 score. The formulas for calculating classification accuracy, sensitivity, specificity, and F1 score are as shown in Equation 6:


(6) $$\begin{array}{c} Acc=\frac{TP+TN}{TP+FP+TN+FN}\\ Sens=\frac{TP}{TP+FN}\\ Spec=\frac{TN}{TN+FP}\\ F1=\frac{2TP}{2TP+FP+FN} \end{array}$$

Here, TP represents true positives, TN represents true negatives, FN represents false negatives, and FP represents false positives.

## 3 Results

### 3.1 Experimental setup

In this study, the deep learning architecture was implemented using the Keras framework with TensorFlow as the backend. Training was conducted on an NVIDIA GeForce RTX 3090 GPU, supporting CUDA 12.0 and cuDNN v8.9. All models in this study maintained consistent training configurations. For the proposed EEG-based depression classification model, the Adam optimization algorithm was used with a batch size of 64 and a learning rate of 0.001. The Adam optimizer adapts the learning rate and combines momentum with adaptive gradient methods, effectively improving the stability of the deep learning model during training. A batch size of 64 was selected to balance computational efficiency and accuracy, enabling effective training acceleration without significantly increasing computational burden. The learning rate of 0.001 was set to ensure smooth convergence during the training process and prevent instability caused by overly large update steps. These hyperparameter configurations were validated through preliminary experiments to ensure the efficiency and stability of model training.

### 3.2 Experimental result

**Figure 4:**
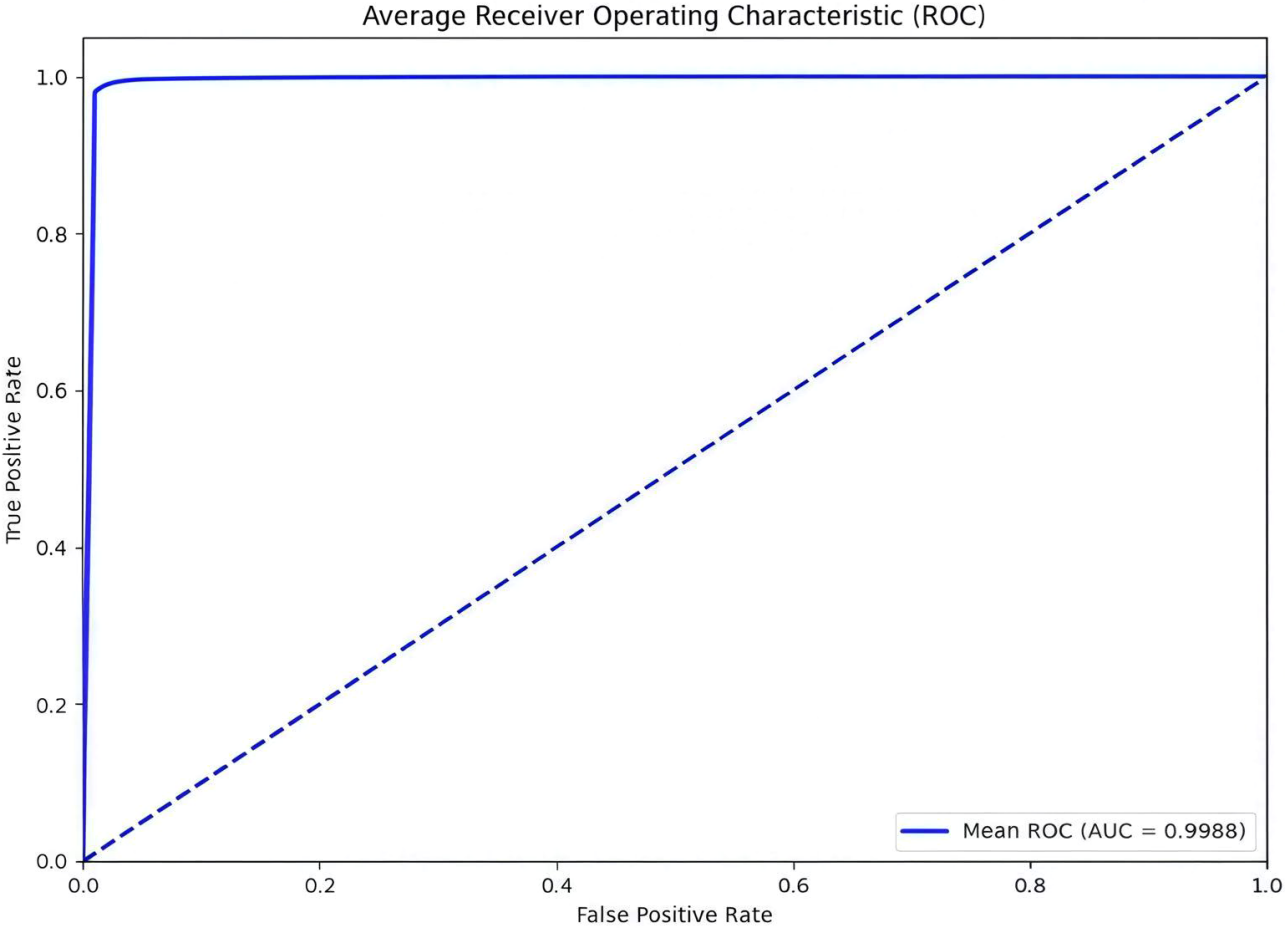
Sensitive feature weighted AUC curve.

**Figure 5:**
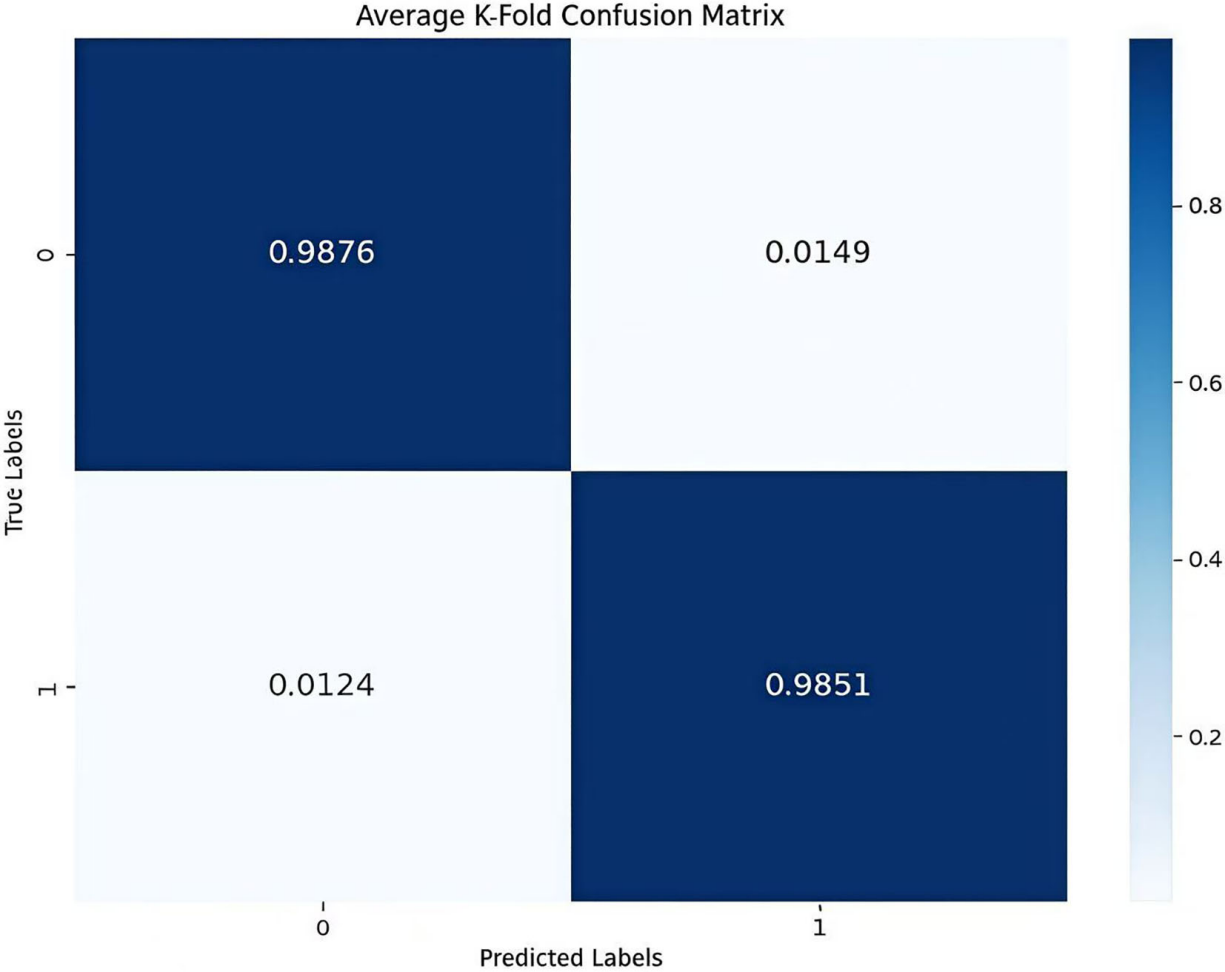
Confusion matrix.

The model uses 10-fold Cross Validation to conduct a binary classification experiment between depression disorder patients and healthy controls. This method involves randomly dividing the dataset into multiple subsets, training and evaluating the model on each split individually, which helps mitigate the risk of overfitting. Additionally, by averaging the various evaluation metrics from the 10 experiments, a more comprehensive and reliable performance assessment can be obtained. In **Figure 4**, the average AUC value reached 99.88%, further indicating that the model demonstrates a strong capability to identify depression disorders. The confusion matrix provides an overall performance metric for the model, detailing the predictions for each category and enabling the evaluation of the classification model’s performance. The confusion matrix for the model AMCCBDep is shown in **Figure 5**, which contains two labels: healthy control group samples and depression patient group samples. The predicted labels are shown on the horizontal axis, while the true labels are displayed on the vertical axis. The diagonal represents the accuracy of the predictions. From the results, it is clear that the proposed method attained high classification accuracy on the dataset.

### 3.3 Detection performance with different numbers of channels

Since the number of EEG channels directly affects the amount of MDD (Major Depressive Disorder) information it contains, this study aims to investigate the impact of different electrode channel counts on the accuracy of MDD recognition. In the experiment, two electrode configurations, 16 channels and 128 channels, were used, and the accuracy, sensitivity, specificity, and F1 score for each configuration were calculated. The related results are shown in **Table 1**. From **Table 1**, it can be seen that the model achieved relatively ideal MDD diagnostic performance with both channel configurations. Specifically, when using 128 channels, the model’s average accuracy reached 99.12%, which is an improvement of 0.44% compared to the 16-channel configuration. Given the considerations of computational efficiency and time performance, in subsequent experiments, unless otherwise specified, the 16-channel configuration will be used as the default input.

**Table 1:** Results for different channels.

Number of electrode channels	ACC(%)	F1(%)	Sens(%)	Spec(%)
16	98.68	98.59	98.64	98.71
128	99.12	99.07	99.00	99.23

### 3.4 Comparison with existing models

As shown in **Table 2**, the AMCCBDep model presented in this study directly processes raw EEG signals, which have undergone basic preprocessing, as input. When evaluated on metrics such as accuracy, F1 score, sensitivity, and specificity, the proposed method outperforms others, demonstrating its ability to effectively capture task-related semantic information in EEG signals while fully leveraging their time-frequency domain characteristics. **Table 1** compares the performance of the proposed approach with other methods using the MODMA dataset.

**Table 2:** Comparison of the performance of AMCCBDep on MODMA datasets with other methods in recent years.

Method	ACC(%)	F1(%)	Sens(%)	Spec(%)
Qayyum et al. (33)	78.2	74.9	–	–
Shen et al. (34)	80.67	81.08	84.13	72.79
Shao et al. (35)	86.7	86.4	–	–
Cui et al. (36)	95.53	95.4	95.12	96.03
Sun et al. (37)	97.22	–	96.57	97.86
Yang et al. (38)	97.43	–	96.99	97.77
Yang et al. (39)	97.56	–	–	–
**AMCCBDep (Ours)**	**98.68**	**98.59**	**98.64**	**98.71**

Bold data indicates the best performing results.

### 3.5 Ablation experiments

To assess the effectiveness of the AMCCBDep model in depression recognition, five distinct model architectures were created to investigate the contribution of each module within AMCCBDep. (i) MsNet: This model uses a multi-scale convolutional structure to extract multi-level frequency features, followed by a fully connected layer for depression detection. (ii) AMPC: This model is based on MsNet for feature extraction, with an added ECA module after each branch of MsNet to adjust feature weights based on channel dependencies for depression detection. (iii) AMPC-BiGRU: This model extracts features using the AMPC-BiGRU module, concatenates the extracted features, and then performs depression detection using a fully connected layer. (iv) AMPC-Conformer: This model incorporates the Conformer module on top of AMPC to extract features, concatenates the extracted features, and then performs depression detection using a fully connected layer. (v) AMCCBDep: This model consists of three main modules: AMPC, Conformer, and BiGRU.

The above five architectures were evaluated using the 10-fold cross-validation method (10-Fold CV), and the experimental results are shown in **Table 3**. As can be seen from the table, the multi-scale convolution MsNet has a relatively low classification accuracy. By comparing the results of MsNet and AMPC, it is found that the ECA module added after each branch adjusts the feature weights based on the dependencies between channels, ensuring that important channel features are enhanced, resulting in a 2.36% increase in classification accuracy. Additionally, AMPC-BiGRU and AMPC-Conformer, compared to AMPC, show that the former effectively fuses multi-scale time-frequency features with the context of EEG time-series extracted by BiGRU, validating the effectiveness of the BiGRU module. The latter demonstrates that the multi-head attention mechanism in the Conformer facilitates the learning of both global and local temporal context features. As a result, the AMCCBDep model effectively captures these global and local features, leading to a significant enhancement in its overall performance.

**Table 3:** Comparison of performance on MODMA datasets.

Model	ACC(%) (mean ± std)	F1(%) (mean ± std)	Sens(%) (mean ± std)	Spec(%) (mean ± std)
MsNet	93.25 ± 0.31	92.64 ± 0.32	90.24 ± 0.42	95.92 ± 0.55
AMPC	95.61 ± 0.68	95.29 ± 0.73	94.40 ± 1.03	96.68 ± 0.74
AMPC-BiGRU	96.89 ± 0.17	96.68 ± 0.18	96.13 ± 0.43	97.58 ± 0.48
AMPC-Conformer	98.31 ± 0.28	98.20 ± 0.29	97.85 ± 0.51	98.72 ± 0.53
AMCCBDep(Our)	98.68 ± 0.45	98.59 ± 0.48	98.64 ± 0.58	98.71 ± 0.47

## 4 Discussion

In this paper, AMCCBDep, demonstrates excellent performance in the task of EEG-based depression recognition by combining the advantages of the AMPC, Conformer, and BiGRU modules. AMCCBDep is a lightweight EEG decoding solution that does not require pretraining, needing only minimal preprocessing steps such as bandpass filtering and normalization, and is not dependent on specific tasks. The AMPC module extracts temporal features using multi-scale convolution kernels and applies depthwise separable convolutions in the spatial dimension (i.e., between channels). After each convolution branch, the ECA mechanism is used to adjust the channel features by applying a weighting scheme. The ECA module captures inter-channel relationships using lightweight convolutions, efficiently enhancing the representational power of channel attention, allowing the model to more accurately recognize and distinguish different EEG signal patterns. The Conformer module further models both global and local temporal dependencies, ensuring the model captures long-range dependencies and local patterns in the EEG signals. The BiGRU module performs bidirectional modeling along the time dimension, capturing bidirectional dependencies in the time series and ultimately outputting feature representations for classification. Therefore, compared to existing CNN-based models (29, 40), the proposed method is capable of learning more discriminative representations.

In the experiments, **Figures 4**, **5** demonstrate that the model achieves high accuracy on public datasets, with evaluation metrics approaching 1, reflecting its excellent generalization capability. Additionally, this paper includes ablation studies that highlight the contribution of each module in the AMCCBDep architecture. Five distinct model configurations were tested to assess the importance of each module in the AMCCBDep structure. The results reveal that the AMCCBDep model effectively captures both global and local features of the modality, leading to a significant improvement in overall performance.

Despite the measures taken, the model still has some limitations. First, while we used standard hyperparameter configurations (such as the Adam optimizer, batch size of 64, and learning rate of 0.001), future work will introduce Grouped Bayesian Optimization (29, 41) to further optimize the hyperparameters and improve the model’s performance. Bayesian optimization can find the optimal configuration with fewer experiments, especially in high-dimensional hyperparameter spaces, thereby enhancing the model’s generalization ability. During training, we employed ten-fold cross-validation, trained for 100 epochs, and used an early stopping mechanism to prevent overfitting, ensuring the model’s stability and generalization ability. The dataset was split into 80% for training and 20% for validation. Secondly, due to significant individual differences in EEG signals, the model’s generalization ability in cross-subject scenarios may be affected, leading to suboptimal classification performance across different individuals. This suggests that future work should explore personalized data processing methods or adopt transfer learning techniques to improve the model’s performance in cross-subject tasks. Future research will focus on applying the model to cross-subject tasks while incorporating visualization techniques (7, 42) to further enhance the model’s interpretability. By visualizing the feature extraction process and the model’s classification decisions, we hope to assist clinical experts in better understanding the internal mechanisms of the model, thereby improving its effectiveness in clinical applications.

## 5 Conclusion

This paper tackles the challenge of inadequate feature extraction and the limitations of convolutional neural networks (CNNs), which are typically restricted to capturing only local features. To address this, a hybrid neural network model for recognizing major depressive disorder (MDD) is proposed, utilizing deep learning techniques. The model integrates the AMPC, Conformer, and BiGRU components to effectively capture complex features from EEG signals. This approach significantly enhances both the classification accuracy and robustness, enabling the model to perform exceptionally well across various time scales and spatial dimensions of EEG data. Additionally, the model streamlines the feature extraction and classification process through an end-to-end framework, minimizing the reliance on manual feature engineering. Experimental findings using the MODMA dataset indicate that the model demonstrates robust performance in analyzing depression through EEG signals. Ablation studies further highlight the crucial role of each module in the AMCCBDep structure, confirming the model’s efficacy and the viability of the proposed depression recognition approach. Despite these achievements, there are still areas for improvement. This study provides a qualitative analysis of mental states, and future research should aim to incorporate quantitative analysis as well.

## Data Availability

The original contributions presented in the study are included in the article/supplementary material. Further inquiries can be directed to the corresponding authors.

## References

[1] Park S-C Kim Y-K. Depression in dsm-5: Changes, controversies, and future directions, (2017) 3–14. 10.1007/978-981-32-9705-0_12 32002930

[2] Evans-Lacko S Aguilar-Gaxiola S Al-Hamzawi A Alonso J Benjet C Bruffaerts R. Socio-economic variations in the mental health treatment gap for people with anxiety, mood, and substance use disorders: results from the who world mental health (wmh) surveys. psychol Med. (2018) 48:1560–71. doi: 10.1017/S0033291717003336 PMC6878971 29173244

[3] Liu J Huang K Zhu B Zhou B Ahmad Harb AK Liu L. Neuropsychological tests in post-operative cognitive dysfunction: methods and applications. Front Psychol. (2021) 12:684307. doi: 10.3389/fpsyg.2021.684307 34149572 PMC8212929

[4] Chen J Lv Y-n Li X-b Xiong J-j Liang H-t Xie L. Urinary metabolite signatures for predicting elderly stroke survivors with depression. Neuropsychiatric Disease and Treatment. (2021) 2021:925–33. doi: 10.2147/NDT.S299835 PMC8007561 33790561

[5] Shen J Zhang X Huang X Wu M Gao J Lu D. An optimal channel selection for eeg-based depression detection via kernel-target alignment. IEEE J Biomed Health Inf. (2020) 25:2545–56. doi: 10.1109/JBHI.2020.3045718 33338023

[6] Rey V Rossetti A Michel P Strambo D Maeder P Dunet V. Perfusion-ct imaging in epileptic seizures. J Neurol. (2018) 265:2972–9. doi: 10.19665/j.issn1001-2400.20230211 30327930

[7] Ke H Wang F Ma H He Z. Adhd identification and its interpretation of functional connectivity using deep self-attention factorization. Knowledge-Based Systems (2022) 250:109082.

[8] Ke H Chen D Yao Q Tang Y Wu J Monaghan J. Deep factor learning for accurate brain neuroimaging data analysis on discrimination for structural mri and functional mri. IEEE/ACM Trans Comput Biol Bioinf. (2023). doi: 10.1109/TCBB.2023.3252577 37028037

[9] Wang F Ke H Ma H Tang Y. Deep wavelet temporal-frequency attention for nonlinear fmri factorization in asd. Pattern Recognition. (2025) 165:111543. doi: 10.1016/j.patcog.2025.111543

[10] Alaei HS Ghoshuni M Vosough I. Directed brain network analysis in anxious and non-anxious depression based on eeg source reconstruction and graph theory. Biomed Signal Process Control. (2023) 83:104666.

[11] Aydın S Cetin FH Uytun MC Babadagi Z Gueven AS Is¸ık Y. Comparison of domain specific connectivity metrics for estimation brain network indices in boys with adhd-c. Biomed Signal Process Control. (2022) 76:103626.

[12] Knott V Mahoney C Kennedy S Evans K. Eeg power, frequency, asymmetry and coherence in male depression. Psychiatry Research: Neuroimaging. (2001) 106:123–40. doi: 10.1016/S0925-4927(00)00080-9 11306251

[13] Liu G-D Li Y-C Zhang W Zhang L. A brief review of artificial intelligence applications and algorithms for psychiatric disorders. Engineering. (2020) 6:462–7. doi: 10.1016/j.eng.2019.06.008

[14] Su Y Zhang Z Cai Q Zhang B Li X. 3dmkdr: 3d multiscale kernels cnn model for depression recognition based on eeg. J Beijing Institute Technol. (2023) 32:230–41. doi: 10.15918/j.jbit1004-0579.2022.096

[15] Chen Y Hu X Xia L. A local-global graph convolutional network for depression recognition using eeg signals. Int J Advanced Comput Sci Appl. (2023) 14. doi: 10.14569/IJACSA.2023.0140720

[16] Zhang B Wei D Yan G Li X Su Y Cai H. Spatial–temporal eeg fusion based on neural network for major depressive disorder detection. Interdiscip Sci.: Computational Life Sciences. (2023) 15:542–59. 10.1007/s12539-023-00567-x PMC10158716 37140772

[17] Costa M Goldberger AL Peng C-K. Multiscale entropy analysis of biological signals. Phys Rev E—Statistical Nonlinear Soft Matter Phys. (2005) 71:021906. doi: 10.1103/PhysRevE.71.021906 15783351

[18] Song Z Deng B Wei X Cai L Yu H Wang J. Scale-specific effects: A report on multiscale analysis of acupunctured eeg in entropy and power. Physica A Stat Mech Appl. (2018) 492:2260–72.

[19] Wang Z Wang Y Hu C Yin Z Song Y. Transformers for eeg-based emotion recognition: A hierarchical spatial information learning model. IEEE Sensors J. (2022) 22:4359–68. doi: 10.1109/JSEN.2022.3144317

[20] Luo G Rao H An P Li Y Hong R Chen W. Exploring adaptive graph topologies and temporal graph networks for eeg-based depression detection Vol. 31. IEEE (2023) p. 3947–57. 10.1109/TNSRE.2023.3320693 37773916

[21] Cai H Yuan Z Gao Y Sun S Li N Tian F. A multi-modal open dataset for mental-disorder analysis. Sci Data. (2022) 9:178. doi: 10.1038/s41597-022-01211-x 35440583 PMC9018722

[22] Shen J Zhang Y Liang H Zhao Z Dong Q Qian K. Exploring the intrinsic features of eeg signals via empirical mode decomposition for depression recognition. IEEE Trans Neural Syst Rehabil Eng. (2022) 31:356–65. doi: 10.1109/TNSRE.2022.3221962 36374871

[23] Do L. “American psychiatric association diagnostic and statistical manual of mental disorders (DSM-IV)”. In: Encyclopedia of child behavior and development (2011). p. 84–5.

[24] Fengwen Z Fanglin S Jing J. Research on depression eeg classification using multi scale convolution combined with transformer. Journal of Xidian University (2023) 51:182–95. doi: 10.19665/j.issn1001-2400.20230211

[25] Akar SA Kara S Agambayev S Bilgic¸ V. Nonlinear analysis of EEGs of patients with major depression during different emotional states. Comput Biol Med. (2015) 67:49–60. 26496702 10.1016/j.compbiomed.2015.09.019

[26] Li X Hu B Sun S Cai H. EEG-based mild depressive detection using feature selection methods and classifiers. Comput Methods Programs Biomed. (2016) 136:151–61. 10.1016/j.cmpb.2016.08.010 27686712

[27] Sun S Li J Chen H Gong T Li X Hu B. A study of resting-state eeg biomarkers for depression recognition. arXiv preprint arXiv:2002.11039 (2020). doi: 10.48550/arXiv.2002.11039

[28] Aydın S. Cross-validated adaboost classification of emotion regulation strategies identified by spectral coherence in resting-state. Neuroinformatics. (2022) 20:627–39. 10.1007/s12021-021-09542-7 34536200

[29] Seal A Bajpai R Agnihotri J Yazidi A Herrera-Viedma E Krejcar O. Deprnet: A deep convolution neural network framework for detecting depression using eeg. IEEE Trans Instrumentation Measurement. (2021) 70:1–13. doi: 10.1109/TIM.19

[30] Jas M Engemann DA Bekhti Y Raimondo F Gramfort A. Autoreject: Automated artifact rejection for meg and eeg data. NeuroImage. (2017) 159:417–29. doi: 10.1016/j.neuroimage.2017.06.030 PMC7243972 28645840

[31] Wang Q Wu B Zhu P Li P Zuo W Hu Q. “Eca-net: Efficient channel attention for deep convolutional neural networks”. In: Proceedings of the IEEE/CVF conference on computer vision and pattern recognition (2020). p. 11534–42.

[32] Gulati A Qin J Chiu C-C Parmar N Zhang Y Yu J. Conformer: Convolutionaugmented transformer for speech recognition. arXiv preprint arXiv:2005.08100 (2020). doi: 10.21437/Interspeech.2020

[33] Qayyum A Razzak I Tanveer M Mazher M Alhaqbani B. High-density electroencephalography and speech signal based deep framework for clinical depression diagnosis. IEEE/ACM Trans Comput Biol Bioinform. (2023) 20:2587–97. 10.1109/TCBB.2023.3257175 37028339

[34] Shen J Li K Liang H Zhao Z Ma Y Wu J. HEMAsNet: A Hemisphere Asymmetry Network Inspired by the Brain for Depression Recognition From Electroencephalogram Signals. IEEE J. Biomed. Health Inform. (2024). 10.1109/JBHI.2024.3404664 38781058

[35] Shao K Wang R Hao Y Hu L Chen M Arno Jacobsen H. “Multimodal physiological signals representation learning via multiscale contrasting for depression recognition,” in Proceedings of the 32nd ACM International Conference on Multimedia. (2024), 5692–701. doi: 10.1145/3664647

[36] Cui W Sun M Dong Q Guo Y Liao X-F Li Y. A multiview sparse dynamic graph convolution-based region-attention feature fusion network for major depressive disorder detection. IEEE Trans Comput Soc Syst. (2023) 11:2691–702. doi: 10.1109/TCSS.2023.3291950

[37] Sun Y Yu J Lu C-T. “Lightk-dsgcn: Depression detection in eegs with lightweight kalman filter-aided dual-stream graph convolutional networks,” in 2023 IEEE International Conference on Bioinformatics and Biomedicine (BIBM). IEEE. (2023), 1429–36.

[38] Yang L Wei X Liu F Zhu X Zhou F. Automatic feature learning model combining functional connectivity network and graph regularization for depression detection. Biomed Signal Process Control. (2023) 82:104520.

[39] Yang L Wang Y Zhu X Yang X Zheng C. A gated temporal-separable attention network for EEG-based depression recognition. Comput Biol Med. (2023) 157:106782. 36931203 10.1016/j.compbiomed.2023.106782

[40] Sharma G Parashar A Joshi AM. Dephnn: A novel hybrid neural network for electroencephalogram (eeg)-based screening of depression. Biomed Signal Process Control. (2021) 66:102393.

[41] Ke H Chen D Shi B Zhang J Liu X Zhang X. Improving brain e-health services via high-performance eeg classification with grouping bayesian optimization. IEEE Trans Serv Computing. (2019) 13:696–708. doi: 10.1109/TSC.4629386

[42] Ke H Chen D Shah T Liu X Zhang X Zhang L. Cloud-aided online eeg classification system for brain healthcare: A case study of depression evaluation with a lightweight cnn. Software: Pract Exp. (2020) 50:596–610. doi: 10.1002/spe.2668

